# Expression of toxic genes in *Methylorubrum extorquens* with a tightly repressed, cumate-inducible promoter

**DOI:** 10.1007/s10482-023-01880-7

**Published:** 2023-09-26

**Authors:** Laura Pöschel, Elisabeth Gehr, Paulina Jordan, Frank Sonntag, Markus Buchhaupt

**Affiliations:** 1 DECHEMA-Forschungsinstitut, Microbial Biotechnology, Theodor-Heuss-Allee 25, 60486 Frankfurt Am Main, Germany; 2 Faculty of Biological Sciences, Goethe University Frankfurt Am Main, Max-Von-Laue-Str. 9, 60438 Frankfurt Am Main, Germany

**Keywords:** Inducible Promoter, *Methylorubrum extorquens* AM1, Alphaproteobacteria, Cumate, Background expression

## Abstract

*Methylorubrum extorquens* is an important model methylotroph and has enormous potential for the development of C1-based microbial cell factories. During strain construction, regulated promoters with a low background expression level are important genetic tools for expression of potentially toxic genes. Here we present an accordingly optimised promoter, which can be used for that purpose. During construction and testing of terpene production strains harbouring a recombinant mevalonate pathway, strong growth defects were observed which made strain development impossible. After isolation and characterisation of suppressor mutants, we discovered a variant of the cumate-inducible promoter P_Q2148_ used in this approach. Deletion of 28 nucleotides resulted in an extremely low background expression level, but also reduced the maximal expression strength to about 30% of the original promoter. This tightly repressed promoter version is a powerful module for controlled expression of potentially toxic genes in *M. extorquens*.

**Supplementary Information:**

The online version contains supplementary material available at 10.1007/s10482-023-01880-7.

## Introduction

Methylotrophs are organisms which use reduced one carbon compounds as sole carbon and energy source. *Methylorubrum extorquens* AM1 serves as model organism for bacterial methylotrophy research since its isolation in 1961 (Peel and Quayle 1961). Furthermore, the organism has gained importance in recent years as a platform organism for C1-biotechnology. Hence, several production routes for bulk and fine chemicals as 1-butanol, 3-hydroxypropionic acid, dicarboxylic acids, mevalonate and α-humulene have been described (Sonntag et al. 2014, 2015a, b; Hu and Lidstrom 2014; Liang et al. 2017; Yang et al. 2017; Schada von Borzyskowski et al. 2018; Lim et al. 2019). Nevertheless, the full potential of *M. extorquens* AM1 as a production platform has not yet been reached (Ochsner et al. 2015). The implementation of new synthetic production routes requires a broad set of molecular tools for DNA introduction, genome manipulation and recombinant gene expression. For many years, only P_*mxaF*_-based expression vectors were used for gene overexpression in *M. extorquens* AM1 (Marx and Lidstrom 2001). The *mxaF* gene, encoding for the large subunit of the methanol dehydrogenase in *M. extorquens*, is highly expressed during methylotrophic growth (Liu et al. 2006) and P_*mxaF*_ is among the strongest known native promoters of *M. extorquens* (Choi et al. 2006)*.* Besides P_*mxaF*_, a variety of synthetic constitutive promoters with different expression strengths have been described (Schada von Borzyskowski et al. 2015). Yet, constitutive expression is not always useful during construction of highly efficient production strains. Inducible and adjustable expression can be necessary to separate growth and production phases or to express toxic genetic constructs. Different inducible promoters with the possibility of tuning the expression level have been described for *M. extorquens* (Choi et al. 2006; Chubiz et al. 2013; Kaczmarczyk et al. 2013; Carrillo et al. 2019; Sathesh-Prabu et al. 2021). In two of the developments, expression levels comparable to or even exceeding those of P_mxaF_ were achieved (Carrillo et al. 2019; Sathesh-Prabu et al. 2021). However, the development of promoters which are suitable for fine-tuned low expression of toxic genes in *M. extorquens* AM1, has not been the subject of studies so far.

In this study, we report on the use of inducible promoters for expression of a gene cluster for *cis*-abienol production in *M. extorquens* AM1. The organism has been described to be especially suited for production of terpenoids via the mevalonic acid (MVA) pathway due to the occurrence of the MVA pathway starting intermediate acetoacetyl-CoA in its primary metabolism (Sonntag et al. 2015a). While in this publication the sesquiterpene α-humulene was the target product, we now aimed at construction of a strain able to synthesise the diterpene alcohol *cis*-abienol. This compound serves as a valuable bioproduct material for the fragrance industry. For production of *cis*-abienol with *M. extorquens* AM1 we planned on using P_Q5_ derivative P_Q2148_ (Kaczmarczyk et al. 2013)_._ The original cumate-inducible promoter system P_Q5_ was designed for expression in *Sphingomonas* species (Kaczmarczyk et al. 2013). For this synthetic promoter, P_syn2_ was combined with control elements of the *Pseudomonas putida* F1 *cym/cmt* system to make it responsive to cumate induction (Eaton 1997; Kaczmarczyk et al. 2013). In the original study, P_syn2_ -32 and -10 regions were exchanged with *M. extorquens* specific sequences. This resulted in P_Q2148_, that can be used to drive gene expression in *M. extorquens.* Surprisingly, in our study P_Q2148_ was not tight enough for using it with the *cis*-abienol synthesis gene cluster and caused strong growth inhibition. Hence, we present P_Q2148_-derivative P_s6_, which is tightly repressible and whose activity is tuneable with different inducer concentrations. With this optimised promoter, we expand the genetic toolkit of *M. extorquens* to promote its use as platform organism for C1 biotechnology.

## Results and discussion

### Terpene production with P_Q2148_-based plasmids

Inducible expression of toxic genes or pathways with toxic intermediates can be essential during the implementation of novel production routes. For instance, previous studies have shown that it is beneficial for terpene production in *M. extorquens* AM1 to increase IPP supply as a metabolic substrate of FPP synthases (Sonntag et al. 2015a). Thereby, constitutive expression of the heterologous MVA pathway had a lethal effect on the cells, and no transformants carrying the corresponding expression plasmid could be isolated in the named study. To overcome this problem, the authors chose the cumate-inducible expression plasmid pQ2148. Although transformation of the construct was successful and product concentrations of the product α-humulene could be increased, the cells still showed a growth defect in the absence of the inducer, which indicates a certain leakiness of the P_Q2148_ promoter (Sonntag et al. 2015a).

**Fig. 1:**
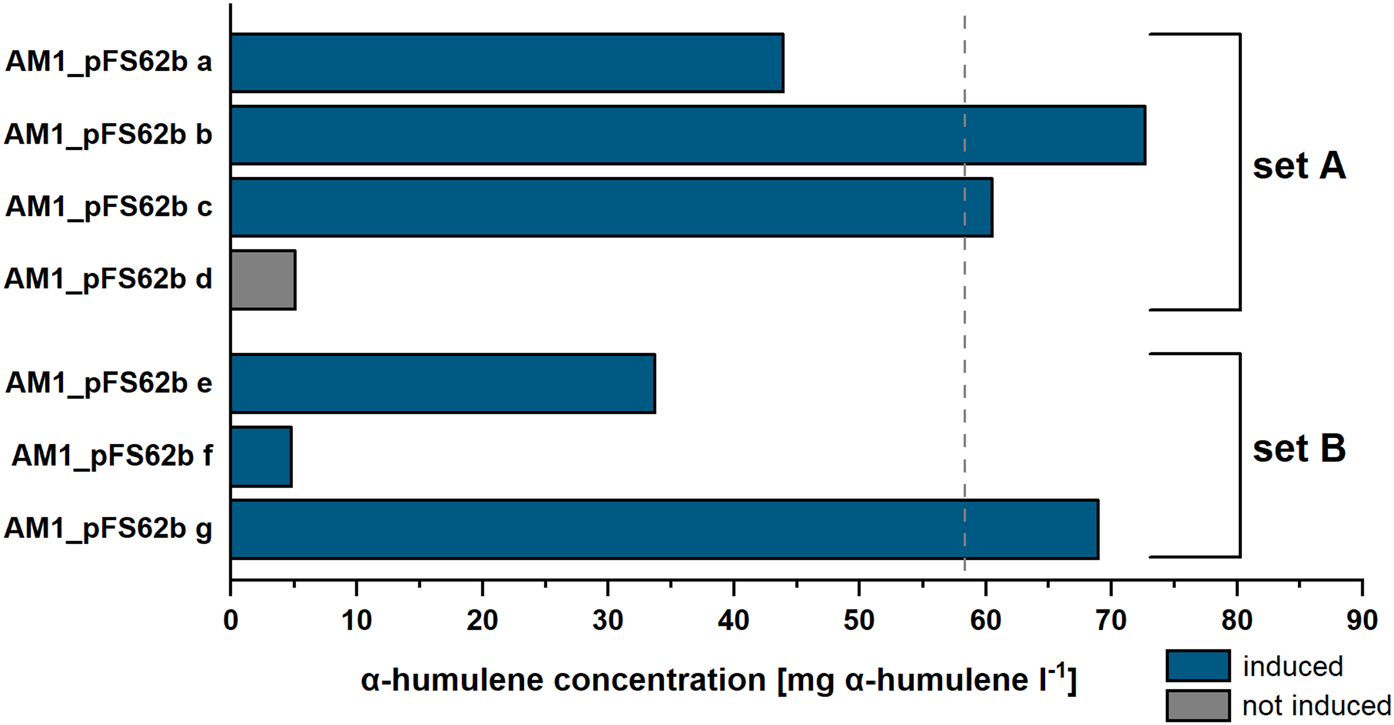
Two datasets (set A and set B) demonstrating α-humulene production with *M. extorquens* AM1 + pFS62b. Different clones, designated a-g, were used in the experiment. The experimental setup was identical to the study Sonntag et al. (2015a). All cultures except the one using clone d were induced with 100 µM cumate. The dashed line marks the previously observed product concentration of 58 mg α-humulene l.^−1^ reported by Sonntag et al. (2015a)

When we attempted to replicate the experiments of the corresponding study (Sonntag et al. 2015a), the amount of α-humulene production surprisingly varied strongly between the different pFS62b-transformants of the AM1 strain (Fig. 1). These strong differences in productivity might be the result of toxic effects of the terpene production gene cluster, which probably caused a strong selection pressure against high carbon flux through the pathway. A non-induced control strain produced 8.7% of the α-humulene titer from the original study (Sonntag et al. 2015a). Another clone showed the same level of production even when induced, probably due to early occurrence of a suppressor mutation. These results indicated that the MVA pathway-encoding plasmid pFS62b exerts some toxic effect on the host strain in the absence of the inducing agent cumate.

These effects became even more evident during an approach to further broaden the terpene product spectrum of *M. extorquens* AM1. The introduction of a *cis*-abienol synthesis operon on plasmid ppjo16 resulted in a very poor transformation efficiency. After transformation of 400 ng of plasmid DNA and six days of incubation, only two colonies appeared on agar plates with medium containing tetracycline, but no cumate, whereas a control transformation of vector pQ2148F yielded over 3000 colonies. This low transformation efficiency rate and the fact that some small colonies appeared on the transformation plates after eight days of incubation led us to the conclusion that the promoter P_Q2148F_ does not tightly regulate the expression of the obviously toxic *cis*-abienol synthesis operon. Streaking out some of the small colonies on a new agar plate resulted in the formation of faster growing strains, which probably contained suppressor mutations and were isolated. The corresponding strains are hereafter referred to as "suppressor mutants".

### Investigation of suppressor mutants

**Fig. 2:**
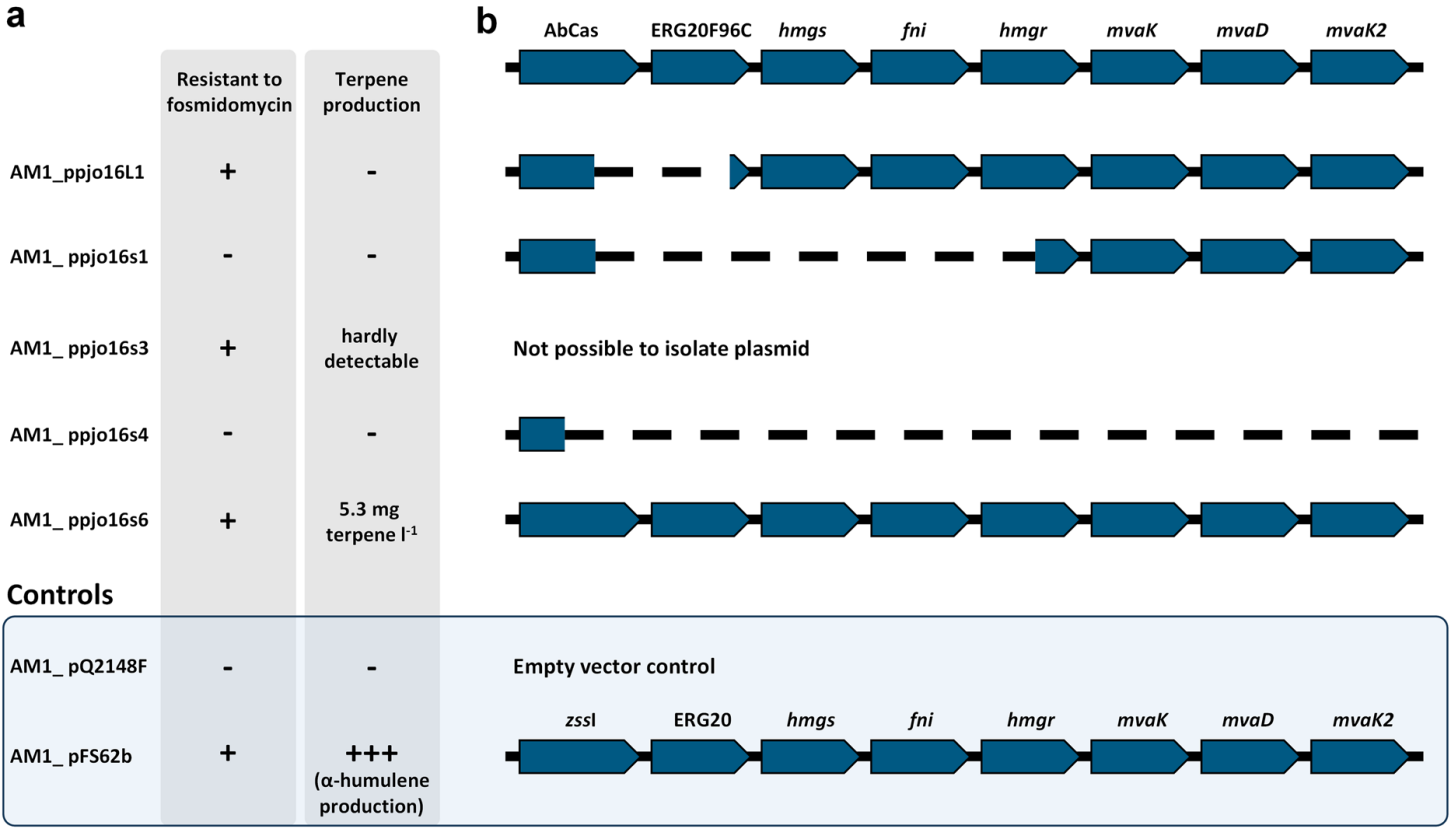
Phenotypes and genotypes of suppressor strains. **a** Phenotypes of suppressor strains regarding fosmidomycin resistance and terpene production. **b** Schematic sequence of terpene synthesis gene cluster (*cis*-abienol synthase gene *AbCAS* and GGPP synthase gene *ERG20F96C*) and MVA gene cluster on plasmid ppjo16 and positions of deletions observed in the plasmid sequences isolated from suppressor mutants

In order to gain knowledge about the underlying cause for growth inhibition mediated by the plasmid ppjo16, the suppressor mutants were thoroughly analysed. First, their sensitivity towards fosmidomycin was tested. Fosmidomycin inhibits the DXP pathway (Shigi 1989; Jomaa et al. 1999), which represents the native terpenoid biosynthesis route of *M. extorquens*. Since IPP and DMAPP supply is crucial for growth of the cells, the suppressor clones were tested for a functional, alternative mevalonate pathway with this assay (Fig. 2A). To further characterise the suppressor mutants, terpene production yields were determined (Fig. 2A), the respective plasmids were isolated and the genes encoding the *cis*-abienol production pathway were sequenced (Fig. 2B). As positive control, an *M. extorquens* AM1 strain harbouring the α-humulene synthesis plasmid pFS62b was used.

The two suppressor mutants unable to grow on fosmidomycin and to produce *cis*-abienol (AM1_ppjo16s1 and AM1_ppjo16s4) were probably able to overcome the plasmid-imposed toxicity by deletion of MVA pathway genes. Additionally, the *cis*-abienol synthase gene *AbCAS* and GGPP synthase gene *ERG20F96C* were partly or completely deleted on the respective plasmids, leading to the inability to produce *cis*-abienol. Only three of the suppressor strains tested were resistant to fosmidomycin, namely AM1_ppjo16L1, AM1_ppjo16s3 and AM1_ppjo16s6. In AM1_ppjo16L1 *AbCAS* and *ERG20F96C* were partly deleted and no *cis*-abienol production was detectable for the respective strain. As the deletion can also affect mRNA stability or translation efficiency of the MVA pathway genes, reduction of MVA pathway flux might be the suppression mechanism also in this mutant. This assumption is supported by the fact that *cis*-abienol itself was found to be not toxic for *M. extorquens* AM1 (Figure S1, Online Resource 1). No plasmid could be isolated from strain AM1_ppjo16s3, so we assume that genomic integration of the entire gene cluster or at least of genes indispensable for *cis*-abienol production has occurred. AM1_ppjo16s6 in fact was able to produce 5.3 mg *cis*-abienol l^−1^ and was resistant to fosmidomycin. The isolated plasmid showed no mutations in any of the genes necessary for *cis*-abienol production or the MVA pathway-encoding genes.

**Fig. 3:**
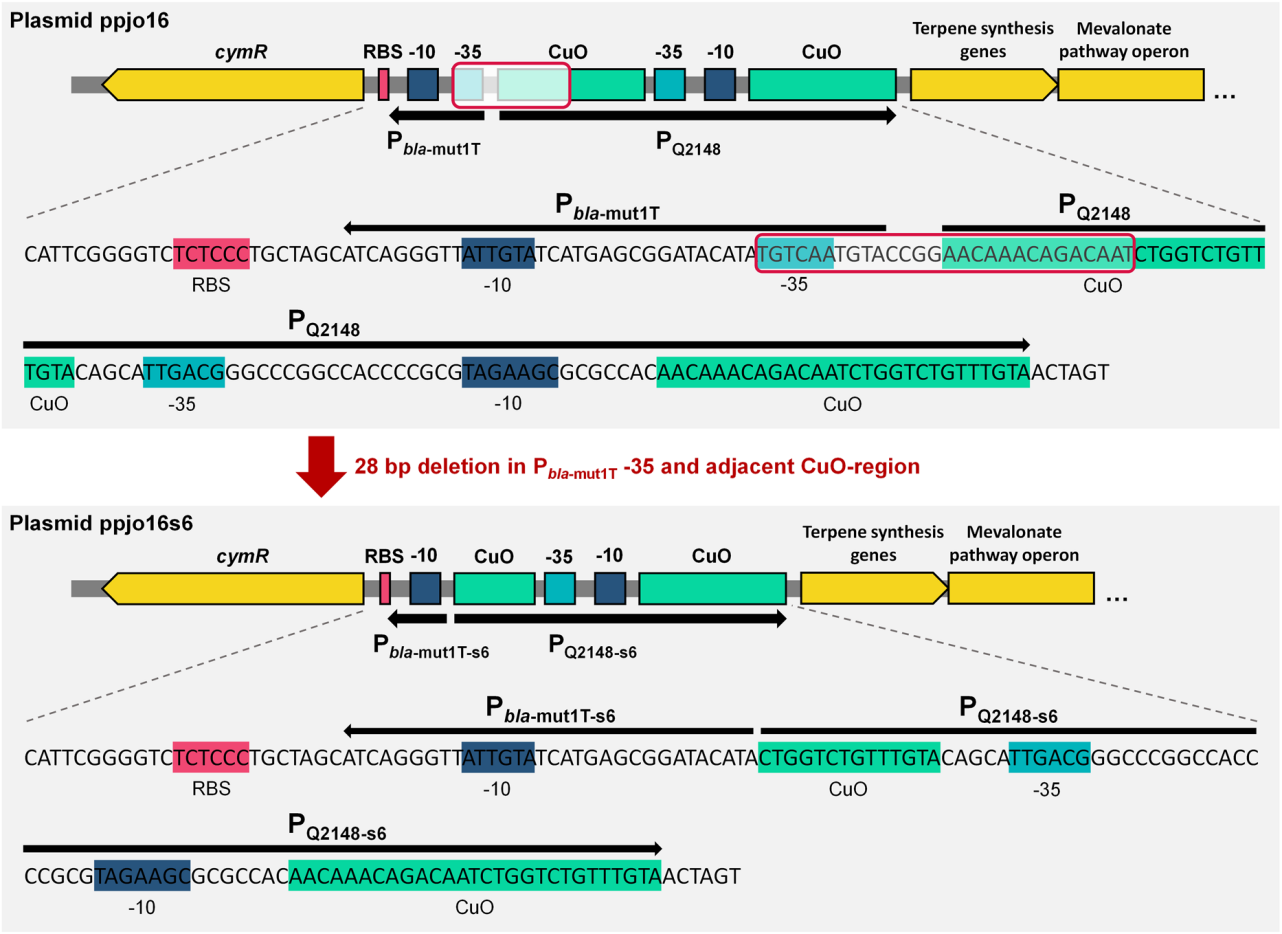
Schematic illustration of *cymR*, the two promoter regions (P_*bla*-mut2T1_ and P_Q2148_) and the terpene synthesis genes on ppjo16 and ppjo16s6. Sequences of coding DNA strands are given in detail for promoter regions. Important features are marked with coloured boxes. P_*bla*-mut2T1_ and P_Q2148_ are indicated by arrows. In ppjo16s6, 28 nucleotides within P_*bla*-mut2T1_ and the close operator region (CuO) in P_Q2148_ are deleted, yielding P_*bla*-mut2T1-s6_ and P_Q2148-s6_, in this study collectively referred to as P_s6_

The original cumate-dependent promoter system P_Q2148_ was already used for inducible gene expression in *M. extorquens* (Kaczmarczyk et al. 2013)*.* In our experiments, expression of the genes encoding the toxic *cis*-abienol production pathway was not tightly repressed by CymR, which is supposed to bind to CuO operator sites as long as no cumate is present. AM1_ppjo16s6 overcame the toxicity of the plasmid while harbouring an intact synthesis operon (Fig. 2B). Sequencing the upstream region of the gene cluster on ppjo16s6 revealed a deletion of 28 nucleotides in the promoter region (Fig. 3). This promoter variant (henceforth P_s6_) lacks parts of the P_*bla*-mut1T_ -35 and the adjacent CuO operator sequence. This modification probably leads to higher expression of the CymR repressor protein or enhanced binding efficiency to the repressor site.

### Characterisation of P_s6_

**Fig. 4:**
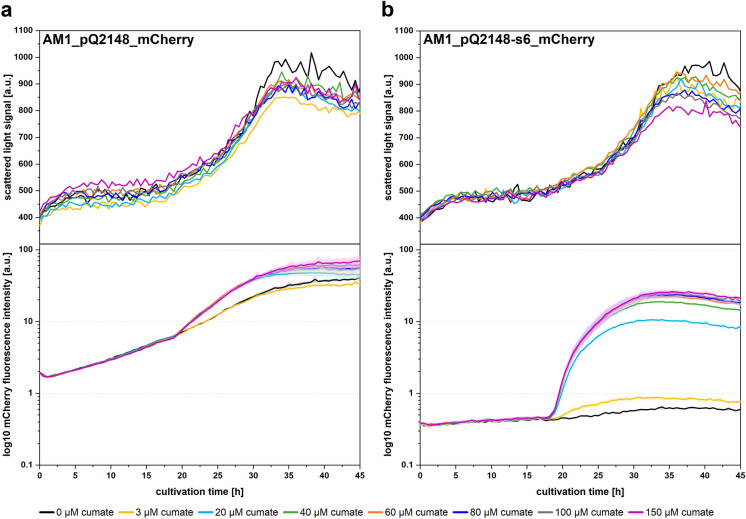
Promoter study of cumate inducible promoter on plasmid pQ2148_mCherry (**a**) and pQ2148_mCherry-s6 (modified promoter P_s6_) (**b**) in a microbioreactor system. *M. extorquens* AM1 cultures containing respective plasmids were induced with cumate after 17.5 h in early exponential growth phase. Top graphs represent cell density measured via scattered light signal at 700 nm, bottom graphs represent mCherry fluorescence signal. Shown datasets are representative for three independent experimental replicates

To further characterise P_s6_, we conducted reporter assays with different cumate concentrations. Therefore, we designed mCherry-reporter constructs pQ2148_mCherry and pQ2148-s6_mCherry and monitored fluorescence of respective *M. extorquens* AM1 transformants in a microbioreactor system. The cumate concentrations we used (up to 150 µM) did not affect cell growth (Fig. 4). A high mCherry signal was already detectable at the begin of cultivation of AM1_pQ2148_mCherry and showed a linear increase in strength even before induction with cumate (Fig. 4A). This confirms that P_Q2148_ in its original confirmation is not as tight as assumed. Induction at 3 µM cumate did not affect fluorescence. Addition of higher inducer levels resulted in induction, although a tuneability with different cumate concentrations was not distinctly evident. In comparison, when investigating AM1_pQ2148-s6_mCherry, the initial fluorescence signal was fivefold lower (Fig. 4B). The signal remained nearly stable until induction (at t_0_: 0.38 ± 0.01; at t_i-1_: 0.45 ± 0.1) and did quickly respond to cumate addition, while tunability was evident. With addition of the highest tested cumate concentration of 150 µM a 40-fold enhanced signal (determined at t_max_ = 33.2 h) compared to the non-induced control could be reached. Moreover, the maximum mCherry fluorescence signal at this high inducer concentration was 54-fold higher (t_max_ = 33.2 h) than immediately before induction (t_i-1_ = 18.3 h).

**Fig. 5:**
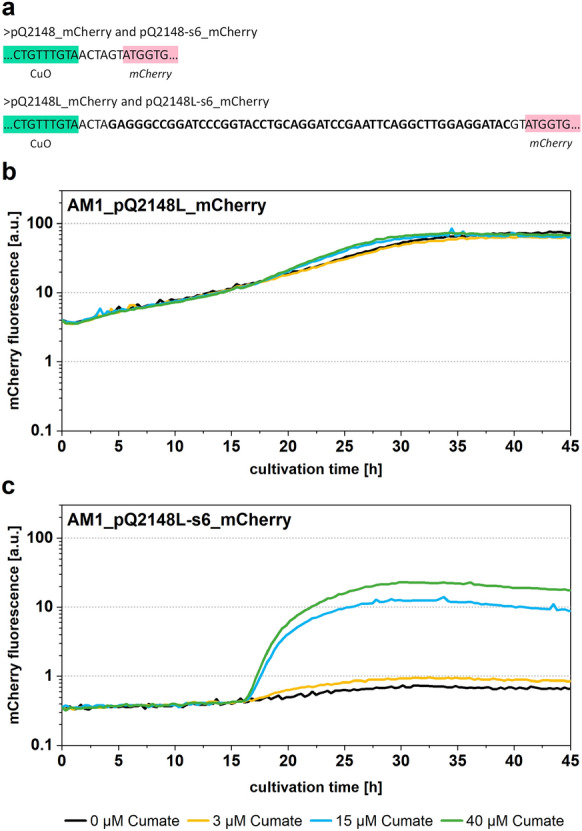
Investigation of P_s6_ promoter variant with the originally described linker region between promoter and controlled gene. **a** Linker region from P_Q2148_*-luxCDABE* (Kaczmarczyk et al. 2013), that was introduced in pQ2148_mCherry and pQ2148-s6_mCherry to yield pQ2148L_mCherry and pQ2148L-s6_mCherry, respectively. Introduced nucleotides are in bold. **b** and **c** Fluorescence study of cumate inducible promoter from plasmid pQ2148L_mCherry (**b**) and pQ2148L_mCherry-s6 (modified promoter P_s6_) (**c**) in a microbioreactor system. Cultures were induced with cumate after 15.5 h in early exponential growth phase. Shown datasets are representative for three independent experimental replicates. Growth was monitored with scattered light signal at 620 nm (data not shown)

P_Q2148_ was shown to be tight and inducible in *M. extorquens* AM1 in previous studies (Kaczmarczyk et al. 2013). Our reporter plasmids carried a different reporter gene, a different selection marker (Tc^r^ instead of Km^r^) and a different linker sequence between promoter and reporter gene due to distinct genesis of the constructs. To validate our observations made with P_Q2148_ and to assure that the sequence differences in the inter-promoter-gene region did not change promoter characteristics, we constructed plasmids pQ2148L_mCherry and pQ2148L-s6_mCherry with the according linker sequence (Fig. 5A). The monitored mCherry fluorescence signals for the new constructs were nearly identical to the previous results. Whereas P_Q2148_ on pQ2148L_mCherry was leaky and promoted mCherry expression even before induction (Fig. 5B), P_s6_ on pQ2148L-s6_mCherry repressed expression without inducer and exhibited quick response to cumate addition with induction at various levels (Fig. 5C).

In either version of the reporter gene constructs, the overall mCherry expression driven by the P_s6_ promoter was considerably lower than with P_Q2148_ (~ 30% of the maximal pQ2148 expression strength). Testing P_s6_ in α-humulene synthesis plasmid led to similar results. Production experiments with the according construct pFS62b-s6 only yielded 28 ± 4 mg α-humulene l^−1^ (three replicates), which is a lower titer compared to experiments with the original plasmid pFS62b. Nevertheless, in the case of *cis*-abienol production, this property of P_s6_ was highly beneficial. The avoidance of pathway-encoding operon expression under non-induced conditions only enabled plasmid transformation and strain cultivation without strong selection for terpene synthesis pathway destruction. P_s6_ furthermore allowed production of *cis*-abienol and will facilitate metabolic engineering approaches towards a more balanced pathway.

## Conclusion

Here, we provide P_s6_, a modified version of the P_Q2148_ promoter (Kaczmarczyk et al. 2013) for cumate inducible gene expression in *M. extorquens*. While P_s6_ is less efficient with regard to strong overexpression, it is a powerful tool for controlled expression of potentially toxic genes or pathways. We successfully demonstrated its application for the development of a *cis*-abienol production strain. Confirmatively, reporter experiments detected essentially no background expression for uninduced constructs. This property makes P_s6_ a valuable addition to the emerging genetic toolbox for *M. extorquens*.

## Material and methods

### Bacterial strains and growth conditions

*Escherichia coli* DH5α (Gibco-BRL, Rockville, USA) was used for cloning and amplification of all plasmids. *E. coli* cultures were grown in LB medium (Bertani 1951) at 37 °C. Liquid minimal medium for *M. extorquens* AM1 (Peel and Quayle 1961) was prepared using 123 mM methanol as previously described (Peyraud et al. 2009) with a CoCl_2_ concentration of 12.6 µM (Kiefer et al. 2009; Sonntag et al. 2014). For preparation of solid growth medium, 1.5% [w/v] agar–agar was added. If necessary, tetracycline was added at a concentration of 10 µg tetracycline hydrochloride ml^−1^ for both *E. coli* and *M. extorquens* AM1 cultures. For cultivation of *M. extorquens* AM1, precultures were grown in test tubes for 48 h at 30 °C and main cultures were subsequently inoculated to an OD_600_ of 0.1. If not stated differently, gene expression was induced after 16 h of cultivation by cumate addition. Cumate (4-isopropylbenzoic acid) was prepared as a 100 mM stock solution in ethanol and diluted prior to use. Fosmidomycin sensitivity of suppressor mutants was tested by streaking out cells on solid medium containing 20 mg fosmidomycin l^−1^. All chemicals used for media preparation were purchased from Carl Roth (Karlsruhe, Germany) or Merck KGaA (Darmstadt, Germany).

### Plasmid construction

All standard cloning procedures were performed in *E. coli* DH5α. Plasmids (see Table S1, Online Resource 1) were constructed as follows. For ppjo16, *cis*-abienol synthase gene *AbCAS* (Zerbe et al. 2012) and GGPP synthase gene *ERG20F96C* (Ignea et al. 2015) were codon optimised and a new RBS sequence was calculated (Salis 2011) and inserted. For the detailed sequence information of genes see international patent WO 2016/142503 (Schrader et al. 2016). Plasmids ppjo16s1, ppjo16s3, ppjo16s4, ppjo16s6 and ppjo16L1 were isolated from AM1_ppjo16 suppressor mutants. To construct pFS62b-s6, a fragment containing P_s6_ was subcloned from ppjo16s6 into pFS62b using NheI and SpeI restriction sites. Reporter plasmids were constructed via Gibson assembly (Gibson et al. 2009): Assembly of PCR product of primers EGe119 and EGe121 on template pTE105_mCherry (Schada von Borzyskowski et al. 2015) and SpeI/EcoRI linearised backbone pQ2148F or ppjo16s6 yielded pQ2148_mCherry or pQ2148-s6_mCherry, respectively. pQ2148L_mCherry was constructed by assembly of PCR products of primers LPoe1 and LPoe2 on template pQ2148 and product of primers LPoe3 and LPoe4 on template pQ2148_mCherry. Accordingly, assembly of PCR products of primers LPoe6 and LPoe7 on template pQ2148_mCherry and product of primers LPoe5 and LPoe8 on template pQ2148_mCherry yielded pQ2148L-s6_mCherry. The sequences of final genetic constructs were confirmed by Sanger sequencing at Eurofins Scientific (Luxembourg, Luxembourg). All used oligonucleotides were purchased from Merck KGaA (Darmstadt, Germany) and are listed in Table S2 (Online Resource 1). PCRs were performed with Q5 Polymerase from NEB (Frankfurt, Germany) according to the manufacturer’s instructions. Subsequently, PCR products were purified with the DNA Clean & Concentrator Kit from Zymo Research Europe (Freiburg, Germany). Transformation of final constructs in *M. extorquens* AM1 was performed as previously described (Toyama et al. 1998).

### Terpene production and analysis

Terpenes produced by *M. extorquens* AM1 strains harbouring respective terpene synthesis plasmids, were extracted in situ with a dodecane overlay as described before (Sonntag et al. 2015a). The analysis of the extracted terpenes was performed on a GC–MS (GC17A with Q5050 mass spectrometer, Shimadzu, Kyoto, Japan) equipped with an Equity 5 column (Supelco, 30 m × 0.25 mm × 0.25 µM) as previously described (Sonntag et al. 2015a). For *cis*-abienol analysis the split ratio was reduced from 1:8 to 1:1 and the overall measuring time was prolonged to 17.5 min. Retention time for *cis*-abienol was 14.1 min. The *cis*-abienol analytical standard was purchased from Toronto Research Chemicals (Toronto, CA).

### Fluorescence assisted promoter studies

For high-resolution measurements of growth curves and mCherry fluorescence signals, cells were cultivated in a BioLector® microbioreactor system (m2p-labs GmbH, Baesweiler, Germany). First, precultures of *M. extorquens* AM1 containing respective reporter plasmids were grown in MeOH minimal medium with 10 µg tetracycline-hydrochloride ml^−1^ for 48 h at 30 °C. Subsequently, 1 ml of fresh medium was inoculated to an OD of 0.1 in 48-well Flowerplates® in the microbioreactor and incubated at 30 °C, 1000 rpm and 85% humidity. The growth was monitored via scattered light signal intensity at 700 nm. The fluorescence signal of mCherry was measured at 580/610 nm [ex/em]. Gene expression was induced by adding 20 µL of cumate stock solutions (solved in ethanol, the final ethanol concentration in the medium was 51 mM).

### Supplementary Information

Below is the link to the electronic supplementary material.

Supplementary file1 (DOCX 72 kb)

## Data Availability

All data generated or analysed during this study are included in this published article and its supplementary information file.
