# Supplementary material for: Expression of toxic genes in Methylorubrum extorquens with a tightly repressed, cumate-inducible promoter
Source: Antonie Van Leeuwenhoek. 2023 Sep 26;116(12):1285–94. doi: 10.1007/s10482-023-01880-7 (PMC10645615; doi:10.1007/s10482-023-01880-7)
Supplement: Supplementary file 1 — Supplementary file1 (DOCX 72 kb) [file 10482_2023_1880_MOESM1_ESM.docx]

**Supplementary Information (****Online Resource 1)**

**Journal:** **Antonie van Leeuwenhoek**

**Expression of toxic genes in *Methylorubrum extorquens* with a tightly repressed, cumate-inducible promoter**

Laura Pöschel^1,2^, Elisabeth Gehr^1^, Paulina Jordan^1^, Frank Sonntag^1^, Markus Buchhaupt^1^*

_1_ DECHEMA-Forschungsinstitut, Microbial Biotechnology, Theodor-Heuss-Allee 25, 60486 Frankfurt am Main, Germany

_2_ Faculty of Biological Sciences, Goethe University Frankfurt am Main, Max-von-Laue-Str. 9, 60438 Frankfurt am Main, Germany

*Corresponding author,

e-mail address: markus.buchhaupt@dechema.de

**Description of content:**

**Table S1** Bacterial strains and plasmids used in this work

**Table S2** Oligonucleotides used in this study

**Figure S1** Tolerance of *M. extorquens* AM1 towards *cis*-abienol dissolved in aqueous phase

**Table S1** Bacterial strains and plasmids used in this work.

| **Name** | **Relevant features/Cloning strategy** | **Application/Source** | **Reference** |
| --- | --- | --- | --- |
| **Bacterial strains** | | | |
| *E. coli* DH5α | F^–^ φ80*lac*ZΔM15, Δ(*lac*ZYA-*arg*F)U169, *rec*A1, *end*A1, *hsd*R17(r_K_^–^, m_K_^+^) *pho*A, *sup*E44, λ^–^, *thi*-1 *gyr*A96 *rel*A1 | Standard cloning applications | ATCC |
| *M. extorquens* AM1 | Cm^R^, gram-negative, facultative methylotrophic, obligate aerobic, α‑proteobacterium |  | Peel and Quayle 1961 |
| **Plasmids** | | | |
| pFS62b | pQ2148F-*zssI-ERG20-hmgs*-MVA | Expression vector for *M. extorquens* AM1 for α‑humulene synthesis | Sonntag et al. 2015 |
| pFS62b-s6 | pQ2148-s6-*zssI-ERG20-hmgs*-MVA | α‑humulene synthesis under P_s6_-control | This work |
| pTE105_mCherry | Tet^R^ | mCherry expression vector for *M. extorquens* AM1 | Schada von Borzyskowski et al. 2015 |
| ppjo16 | pQ2148F-*AbCAS‑ERG20F96C*‑MVA, *AbCAS* (Zerbe et al. 2012) and *ERG20F96C* (Ignea et al. 2015) were codon optimized and a new RBS^a^ was inserted. For detailed sequence information see international patent WO 2016/142503 (Schrader et al.) | *cis*-abienol production plasmid | This work |
| ppjo16s1 | pQ2148F_*AbCAS*(mut.)*_*MVA(mut.) | Isolated from ppjo16-suppressor mutant | This work |
| ppjo16s3 | pQ2148F_*AbCAS_ERG20F96C*_MVA | Isolated from ppjo16-suppressor mutant | This work |
| ppjo16s4 | pQ2148F_*AbCAS*(mut.) | Isolated from ppjo16-suppressor mutant | This work |
| ppjo16s6 | pQ2148F_*AbCAS_ERG20F96C*_MVA (mutated P_Q2148_) | Isolated from ppjo16-suppressor mutant | This work |
| ppjo16L1 | pQ2148F_*AbCAS*(mut.)*_ERG20F96C*  (mut.)_MVA | Isolated from ppjo16-suppressor mutant | This work |
| pQ2148 | P_Q2148_, Tet^R^, oriT, pBR322ori | Expression vector for *M. extorquens* harboring cumate inducible promoter | Kaczmarczyk et al. 2013 |
| pQ2148F | pQ2148 with adapted multiple cloning site, Tet^R^, oriT, pBR322ori | Expression vector for *M. extorquens* harboring cumate inducible promoter | Sonntag et al. 2015 |
| pQ2148_mCherry | P_Q2148_, mCherry, Tet^R^, oriT, pBR322ori | mCherry reporter plasmid for P_Q2148_ | This work |
| pQ2148-s6_mCherry | P_s6_, mCherry, Tet^R^, oriT, pBR322ori | mCherry reporter plasmid for P_s6_ | This work |
|  |  |  |  |
| pQ2148L_mCherry | P_Q2148_, mCherry, Tet^R^, oriT, pBR322ori, contains linker region of pQ2148-lux (Kaczmarczyk et al. 2013) | mCherry reporter plasmid for P_Q2148_ with same GOI-upstream sequence like pQ2148-lux (Kaczmarczyk et al. 2013) | This work |
| pQ2148L-s6_mCherry | P_s6_, mCherry, Tet^R^, oriT, pBR322ori, contains linker region of pQ2148-lux (Kaczmarczyk et al. 2013) | mCherry reporter plasmid for P_s6_ with same GOI-upstream sequence like pQ2148-lux (Kaczmarczyk et al. 2013) | This work |

^a^ Optimization of RBS sequences was done with the RBS Calculator (Salis 2011)

**Table S2** Oligonucleotides used in this study^a^.

| EGe119 | ACAATCTGGTCTGTTTGTAACTAGTATGGTGAGCAAGGGCGAG | Construction of pQ2148F_mCherry and pQ2148F-s6_mCherry |
| --- | --- | --- |
| EGe121 | TTGTAAAACGACGGCCAGTGAATTCTTACTTGTACAGCTCGTCCATGCC |  |
| LPoe1 | AGCCTGAATTCGGATCCTGCAGGTACCGGGATCCGGCCCTCTAGTTACAAACAGACCAGATTGTCTGTTTGTTGTGGCGCGCTTCTAC | Construction of pQ2148_mCherry and pQ2148-s6_mCherry |
| LPoe2 | CATGGACGAGCTGTACAAGTAAGAATTCACTGGCCGTCGTTTTACAACGTCGTGACTGG |  |
| LPoe3 | CGGTACCTGCAGGATCCGAATTCAGGCTTGGAGGATACGTATGGTGAGCAAGGGCGAGG |  |
| LPoe4 | GTAAAACGACGGCCAGTGAATTCTTACTT |  |
| LPoe5 | GTATCATGAGCGGATACATACTGGTCTGTTTGTACAGCATTGACG |  |
| LPoe6 | TATGTATCCGCTCATGATACAATAACCCTGATGC |  |
| LPoe7 | GCCTCGCGCGGGATTTTCTT |  |
| LPoe8 | CTGTTCACCACGCGCAACAAG |  |
| PJo113 | TTCGGCGACATGATGAC | Sequencing of constructs |

^a^ For oligonucleotides and PCR templates used for construction of ppjo16, see international patent WO 2016/142503 (Schrader et al.)





**Fig. S1** Tolerance of *M. extorquens* AM1 towards *cis*-abienol. Maximum growth rates (μ_max_) in medium without *cis*-abienol were compared to growth rates (μ) with different *cis*-abienol concentrations dissolved in aqueous phase. Three to four independent replicates were measured. Error bars represent standard deviations

**References**

Ignea C, Trikka FA, Nikolaidis AK, et al (2015) Efficient diterpene production in yeast by engineering Erg20p into a geranylgeranyl diphosphate synthase. Metab Eng 27:65–75. https://doi.org/10.1016/j.ymben.2014.10.008

Kaczmarczyk A, Vorholt JA, Francez-Charlot A (2013) Supplemental material Cumate-inducible gene expression system for sphingomonads and other Alphaproteobacteria. Appl Environ Microbiol 79:6795–6802. https://doi.org/10.1128/AEM.02296-13

Peel D, Quayle JR (1961) Microbial growth on C_1_ compounds. 1. Isolation and characterization of *Pseudomonas* AM 1. Biochem J 81:465–469. https://doi.org/10.1042/bj0810465

Salis HM (2011) The ribosome binding site calculator. Methods Enzymol 498:19–42. https://doi.org/10.1016/B978-0-12-385120-8.00002-4

Schada von Borzyskowski L, Remus-Emsermann M, Weishaupt R, et al (2015) A Set of Versatile Brick Vectors and Promoters for the Assembly, Expression, and Integration of Synthetic Operons in *Methylobacterium extorquens* AM1 and Other Alphaproteobacteria. ACS Synth Biol 4:430–443. https://doi.org/10.1021/sb500221v

Schrader J, Buchhaupt M, Sonntag F, et al PROCESS FOR DE NOVO MICROBIAL SYNTHESIS OF TERPENES. WO 2016/142503 A1, 2016

Sonntag F, Kroner C, Lubuta P, et al (2015) Engineering *Methylobacterium extorquens* for de novo synthesis of the sesquiterpenoid α-humulene from methanol. Metab Eng 32:82–94. https://doi.org/10.1016/j.ymben.2015.09.004

Zerbe P, Chiang A, Yuen M, et al (2012) Bifunctional *cis*-Abienol Synthase from *Abies balsamea* Discovered by Transcriptome Sequencing and Its Implications for Diterpenoid Fragrance Production. J Biol Chem 287:12121–12131. https://doi.org/10.1074/jbc.M111.317669
